# Knockout of *Arabidopsis* Serotonin *N*-Acetyltransferase-2 Reduces Melatonin Levels and Delays Flowering

**DOI:** 10.3390/biom9110712

**Published:** 2019-11-06

**Authors:** Hyoung Yool Lee, Kyungjin Lee, Kyoungwhan Back

**Affiliations:** Division of Food Technology and Biotechnology, College of Agriculture and Life Sciences, Chonnam National University, Gwangju 61186, Korea; xanthine@naver.com (H.Y.L.); nicekj7@hanmail.net (K.L.)

**Keywords:** *Arabidopsis*, recombinant SNAT2, flowering, melatonin, *snat2* knockout

## Abstract

Melatonin plays roles in both plant growth and defense. Serotonin *N*-acetyltransferase (SNAT) catalyzes formation of *N*-acetylserotonin (NAS) from serotonin. Plants contain two *SNAT* isogenes, which exhibit low-level amino acid homology. We studied the *Arabidopsis* *thaliana* *SNAT2* (*AtSNAT2*) gene; we prepared recombinant SNAT2 protein and characterized a *snat2* knockout mutant. The SNAT2 protein exhibited 27% amino acid homology with SNAT1; the *K*_m_ was 232 μM and the *V*_max_ was 2160 pmol/min/mg protein. Melatonin inhibited SNAT enzyme activity in vitro. *SNAT2* mRNA was abundantly expressed in flowers; the melatonin content of flowers of the *snat2* mutant was significantly less than that of wild-type flowers. The mutant exhibited delayed flowering and reductions in leaf area and biomass compared to the wild type. Delayed flowering was attributable to reductions in the expression levels of the gibberellin biosynthetic genes *ent-kaurene synthase* (*KS*) and *FLOWERING LOCUS T* (*FT*).

## 1. Introduction

The recent identification of an *Arabidopsis* phytomelatonin receptor implied that melatonin may serve as a plant hormone [1,2]. Melatonin plays many biological roles in plants, being involved in leaf senescence [3], quality control of the endoplasmic reticulum [4], plant growth [5,6], secondary metabolite synthesis [7,8], somatic embryogenesis [9], stomatal closure [1], skotomorphogenesis [10], and leaf development [11]. Melatonin plays roles in plant responses to both biotic and abiotic stressors [12,13,14], mediating tolerance to drought [15], high salt levels [16], high temperatures [17], cold conditions [18], heavy metals [19,20,21,22], and pathogens [23,24].

All plant melatonin biosynthetic genes have been cloned [25]. The genes encoding the last two steps in melatonin biosynthesis are serotonin *N*-acetyltransferase (*SNAT*) and *N*-acetylserotonin *O*-methyltransferase (*ASMT*). Plants contain two *SNAT* isogenes [25]; the rice genome contains three copies of *ASMT* [26]. *SN*A*T* plays important roles in both melatonin function and biosynthesis. Suppression of rice *SNAT2* decreased levels of brassinosteroids (plant hormones) and induced a dwarf phenotype, but, suppression of rice *SNAT1* did not result in such effects, although melatonin levels were similarly decreased [10]. The in vitro enzyme kinetics of rice SNAT1 and SNAT2 differed [25]. The *SNAT1* genes of various plants (and cyanobacteria) have been cloned and characterized [27,28,29], but *SNAT2* has been cloned only from rice [30] and grapevine [31].

Although many physiological functions played by melatonin have been reported, there has not been any molecular genetic study on the effect of flowering. Plant flowering is initiated by physical factors such as the photoperiod and vernalization, and by gibberellic acid (GA; a hormone; [32]). The key photoperiod-regulated flowering gene is the night-labile *CONSTANT* (*CO*), a zinc finger protein that activates *FLOWERING LOCUS T* (*FT*), which in turn promotes floral induction. Vernalization accelerates flowering by decreasing the expression of *FLOWERING LOCUS C* (*FLC*), which encodes a MADS-box transcription factor that represses *FT*. In addition to the above physical factors, GA also directly or indirectly controls FT expression.

Here, we cloned *Arabidopsis thaliana SNAT2* and explored the kinetics of the recombinant protein. We investigated an *SNAT2* knockout mutant (*snat2*) in which decreased melatonin levels delayed flowering and retarded growth.

## 2. Materials and Methods

### 2.1. Plant Materials and Growth

*Arabidopsis thaliana* wild-type Col-0 and the knockout snat2 mutant line (SALK_062388) were grown at 22 °C under 60% relative humidity, a 14 h photoperiod, and a photon flux density of 50 µmol m^−2^ s^−1^. The *Arabidopsis* snat2 line (with a T-DNA insert in SALK_062388) was obtained from the Arabidopsis Biological Resource Center (Ohio State University, Columbus, OH, USA). The line was genotyped using the T-DNA express: *Arabidopsis* gene mapping tool (http://signal.salk.edu/). Tobacco plants (*Nicotiana benthamiana*) were grown under a 16 h light (28 °C)/8 h dark (22 °C) photoperiod featuring white light (100 μmol photons m^−2^ s^−1^). Flowering times were defined by the days to bolting and the numbers of rosette leaves at bolting (n = 20). Leaf area was measured using Fuji ImageJ software [33]. Rosette leaves were photographed adjacent to a ruler; pixels were then converted to metric units. All data were processed with the aid of Microsoft Excel 2010. All experiments were performed in triplicate and were repeated at least three times.

### 2.2. Homology and Phylogenetic Analysis of AtSNAT2

Amino acid sequence homology was explored with the aid of the BLASTp tool; we interrogated the nonredundant, protein sequence databases of the National Center for Biotechnology Information (http://www.ncbi.nlm.nih.gov/). BLAST-Explorer was used to generate sequence alignments and phylogenetic trees [34].

### 2.3. Cloning of AtSNAT2 and Purification of Recombinant AtSNAT2 Protein from Escherichia Coli

Total RNA of 3-week-old *Arabidopsis* leaves was isolated using a NucleoSpin RNA plant kit (Macherey-Nagel, Duren, Germany) and 1 µg thereof was reverse-transcribed using RevertAid transcriptase (Thermo Scientific Fermentas, St. Leon-Ro, Germany) after addition of 500 ng of oligo(dT)18 primer, at 42 °C for 1 h. The cDNA obtained (0.2 µL) served as the template for PCR-based cloning. Reverse transcription (RT)-PCR was employed to clone full-length *AtSNAT2* (GenBank accession number BT005218) using the primer pair 5′-AAA AAG CAG GCT CCA TGT TTC TCG GAG GCA-3′ and 5′-AGA AAG CTG GGT TTA TTT CTT GTT TCT CTG-3′. The initial *AtSNAT2* PCR product was cloned into the T&A vector (RBC Bioscience, New Taipei City, Taiwan) and the integrity thereof confirmed via sequencing (Bioneer, Daejeon, Korea). Prior to AtSNAT2 expression and purification, the initial RT-PCR product was amplified using a primer pair containing *attB* recombination sequences (forward primer, 5′-GGG GAC AAG TTT GTA CAA AAA AGC AGG CT-3′; reverse primer, 5′-GGG GAC CAC TTT GTA CAA GAA AGC TGG GT-3′). The resulting *AtSNAT2* PCR product was cloned via the Gateway recombination reaction into the pDONR221 vector (Invitrogen, Carlsbad, CA, USA), and then into the destination vector pET300/NT-DEST (Invitrogen). The pET300-AtSNAT2 plasmid was transformed into *E. coli* strain BL21(DE3) (Invitrogen). An aliquot (10 mL) of an overnight culture grown in the presence of ampicillin (50 mg/L) was inoculated into 100 mL of Terrific Broth [20 g/L Bacto-tryptone, 24 g/L Bacto-yeast extract, glycerol 4 mL/L, and phosphate buffer (0.017 M KH_2_PO_4_ and 0.072 M K_2_HPO_4_)] containing 50 mg/L ampicillin and incubated at 37 ℃ until the optical density at 600 nm attained 1.0. After addition of isopropyl-β-D-thiogalactopyranoside (IPTG; Sigma, St. Louis, MO, USA) to 1 mM, the culture was grown at 28 ℃ with shaking at 180 rpm for 5 h. The protein was purified via affinity (Ni^2+^) chromatography according to the recommendations of the kit manufacturer (Qiagen, Tokyo, Japan).

### 2.4. AtSNAT Enzyme Kinetics

Purified recombinant AtSNAT2 protein was incubated in a final volume of 100 µL with 0.5 mM serotonin or 5-methoxytryptamine (5-MT), and 0.5 mM acetyl-CoA, in 100 mM potassium phosphate (at pH 7.8 or other pH values) at 45 °C (or other temperatures) for 1 h. Reaction products including *N*-acetylserotonin (NAS) and melatonin were identified with the aid of high-performance liquid chromatography (HPLC). In brief, each reaction was terminated by addition of 20 µL acetic acid and 30 µL methanol and 10 µL aliquots were subjected to reverse-phase HPLC (Waters, Milford, MA, USA) on a SunFire C18 column (Waters; 4.6 × 150 mm) eluted using an isocratic profile of 6% (v/v) methanol for NAS or 10% (v/v) methanol (both in water with 0.3% [v/v] trifluoroacetic acid) at a flow rate of 1 mL/min. Compounds were detected via absorbance at 280 nm. The substrate affinity (*K*_m_) and maximum reaction rate (*V*_max_) were calculated using Lineweaver–Burk plots. Protein concentrations were determined using the Bradford method employing a protein-specific dye (Bio-Rad, Hercules, CA, USA). All analyses were performed in triplicate.

### 2.5. Subcellular Localization of AtSNAT2

We used the pER-mCherry vector [the kind gift of Dr. H.G. Kang (Texas State University, San Marcos, TX, USA)] to localize the AtSNAT2 protein. Full-length *AtSNAT2* was PCR-amplified using a primer set containing *Asc*I sites (GGCGCGCC). The resulting product was cloned into the T&A vector (RBC Bioscience) and the *AtSNAT2* insert was digested with *Asc*I and cloned into the *Asc*I site of the binary vector, pER8-mCherry, containing the estrogen-inducible XVE promoter. The pER8-AtSNAT2-mCherry plasmid was transferred into *Agrobacterium tumefaciens* strain GV2260 using the freeze–thaw method. A confocal microscope (TCS-SP5; Leica, Wetzlar, Germany) was used to evaluate transient within-cell protein expression, as described previously [35].

### 2.6. Total RNA Isolation and Reverse Transcription/Polymerase Chain Reaction

Total plant RNA was isolated using a NucleoSpin RNA plant kit (Macherey-Nagel, Duren, Germany). Semi-quantitative reverse transcription (RT)-PCR analyses were performed as described previously [21]. The primer sequences used for PCR amplification were as follows: *SNAT1* (forward, 5′-GCC AAG GAG ACC GTT AGT GA-3′, reverse, 5′-CTT TGG GTA CCA AAA CAT GCC-3′); *SNAT2* (forward, 5′-ATG TTT CTC GGA GGC ACA AT-3′, reverse, 5′-ATT TCT TGT TTC TCT GTT TGC GT-3′); *CCA1* (forward, 5′-GTT GCA GCT GCT AGT GCT TG-3′, reverse 5′-CAA AGG CCT CAA AAG AAA AAG A-3′); *TOC1* (forward, 5′-TCT CCG GTG ACT TTT GTT GA-3′, reverse 5′-TGG CCA AAT CAG AAC TAG GG-3′); *KS* (forward, 5′-CCA AGT TGA TCT GGC AGG TA-3′, reverse 5′- TTG TCT CCT AAA ATC AAT TTT CCT C-3′); *MYB33* (forward, 5′-TTG TTC TTG GAG CAA CA TGC-3′, reverse 5′-TGC TTG GCA GTT GCT AGT C-3′); *FT* (forward, 5′-GGT GGA GAA GAC CTC AGG AA-3′, reverse 5′-CTC ATT TTC CTC CCC CTC TC-3′); *BIP2* (forward, 5′-GCA GGA GGA GAA TCA TCG AC-3′, reverse 5′-AAA GAG AAC GTC CAG GGA GA-3′); and *PR1* (forward, 5′-CTA TAT AAG GAA GTT CAT TT-3′, reverse 5′-CTA TAT AAG GAA GTT CAT TT-3′). *EF-1a* transcript levels served to normalize all signals (forward, 5′-TGG TGA CGC TGG TAT GGT TA-3′, reverse 5′- CAT CAT TTG GCA CCC TTC TT-3′).

### 2.7. Melatonin Quantification

Arabidopsis leaves (0.2 g) were ground in liquid nitrogen and thoroughly homogenized in 2 mL chloroform. After centrifugation at 12,000 × g for 10 min, the supernatants were decanted and evaporated to dryness, and the residues were dissolved in 1 mL amounts of methanol; 10 µL aliquots were used for melatonin quantitation via liquid chromatography/dual mass spectrometry (LC-MS/MS; Shimadzu, Kyoto, Japan) as described previously [36]. A mass transition from m/z 233.1 to m/z 174.0 identified melatonin; the retention time was 3.65 min. AB Sciex Analyst software (version 1.5.2) was used for data integration.

### 2.8. Data Analysis

We used the Student’s *t*-test of SigmaPlot ver. 10 (Systat Software, Point Richmond, CA, USA). *p* < 0.05 was considered to indicate statistical significance.

## 3. Results

### 3.1. Cloning and Phylogeny of AtSNAT2

**Figure 1:**
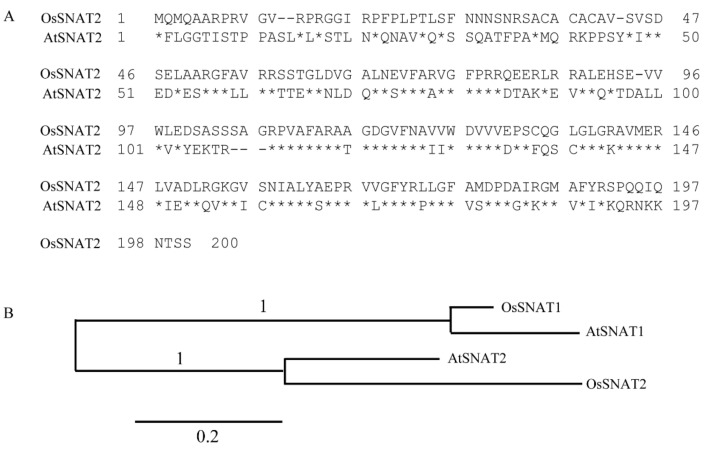
Amino acid comparisons among, and the phylogenetic trees of, *SNAT* isogenes. (**A**) Predicted amino acid sequences of OsSNAT2 and AtSNAT2. (**B**) Phylogenetic analyses of rice and *Arabidopsis SNAT* isogenes. Identity is denoted by stars. Gaps are noted as dashes. Scale bar: substitution value of 0.2/site. Phylogenetic analysis was performed with the aid of BLAST-Explorer (www.phylogeny.fr). The GenBank accession numbers are *OsSNAT1* (AK059369), *OsSNAT2* (AK068156), *AtSNAT1* (At1g32070), and *AtSNAT2* (At1g26220; BT005218).

SNAT is the penultimate enzyme of the melatonin biosynthetic pathway; plants contain two *SNAT* isogenes [30]. Plant *SNAT1* genes have been relatively well studied [25], but the *SNAT2* genes of only rice [10] and grapevine [31] have been evaluated. No plant *SNAT2* knockout mutant has yet been investigated. Here, we cloned the rice *SNAT2* homolog of *Arabidopsis* (*AtSNAT2*). The amino acid sequences of the rice SNAT2 and AtSNAT2 were 48% identical (Figure 1A). Plant SNAT1 and SNAT2 share an amino acid identity level <27%. The two *AtSNAT* isogenes belong to different clades, implying that they arose via independent evolution rather than gene duplication (Figure 1B).

### 3.2. Subcellular Location of AtSNAT2

**Figure 2:**
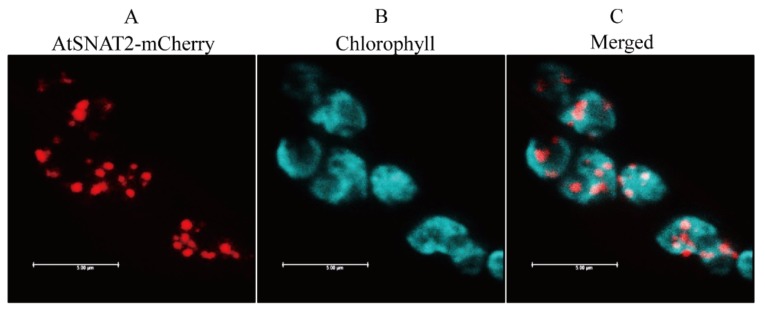
Subcellular localization of AtSNAT2. (**A**) Red fluorescence of AtSNAT2-mCherry. (**B**) Cyan fluorescence of chlorophyll. (**C**) Merged fluorescent images (A+B). Tobacco leaves were infiltrated with *Agrobacterium tumefaciens* GV2260 expressing the XVG-inducible AtSNAT2-mCherry fusion protein. Bars: 5 μm.

To evaluate whether AtSNAT2 is also expressed in chloroplasts, we transiently expressed the AtSNAT2-mCherry fusion protein in tobacco leaves. Figure 2 shows that the protein localized to chloroplasts; the chlorophyll and mCherry fluorescences overlapped precisely. Thus, AtSNAT2 is expressed in chloroplasts, as are other plant SNAT proteins [30,31,35].

### 3.3. AtSNAT2 Enzyme Kinetics

**Figure 3:**
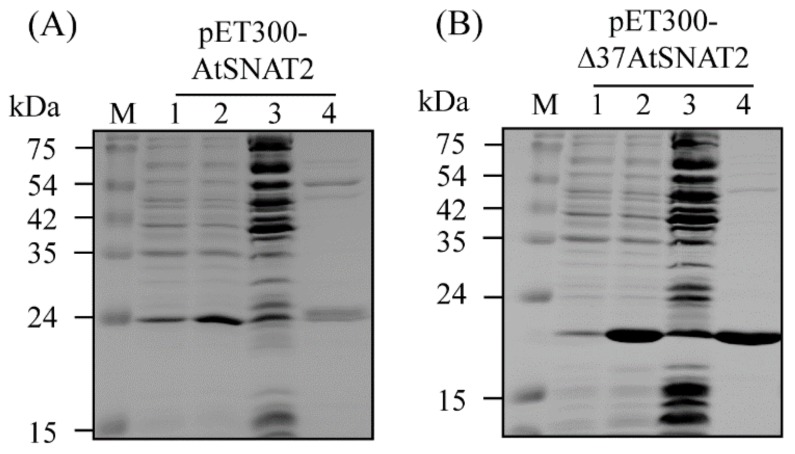
Affinity purification of AtSNAT2 from *Escherichia coli*. (**A**) Purification of the complete AtSNAT2 protein. (**B**) Purification of the protein lacking the N-terminal 37 amino acids. *E. coli* BL21 (DE3) cells harboring pET300-AtSNAT2 and pET300-Δ37AtSNAT2 were employed for protein purification. M, molecular mass standards; lane 1, total proteins of 15 µL aliquots of bacterial cultures grown in the absence of IPTG; lane 2, total proteins of 15 µL aliquots of bacterial cultures grown in the presence of IPTG; lane 3, 20 µg of soluble proteins; lane 4, 10 µg of protein purified via affinity chromatography. Protein samples were separated on 12% (w/v) SDS-PAGE gels and stained with Coomassie blue.

We expressed two *AtSNAT2* gene constructs in *E. coli*; the chloroplast-targeting sequence inhibits heterologous protein expression in that bacterium [37]. We thus expressed both full-length AtSNAT2 and an N-terminal-37-amino acid-truncated protein. TargetP analysis predicted the presence of an N-terminal 37-amino acid transit sequence [38]. We purified both recombinant proteins via Ni^2+^ affinity chromatography; both featured N-terminal hexa-histidine fusions (Figure 3). IPTG enhanced the expression levels of both proteins, but the extent of inducibility and the expression levels of the N-terminal-truncated Δ37AtSNAT2 were greater than those of the full-length protein.

**Figure 4:**
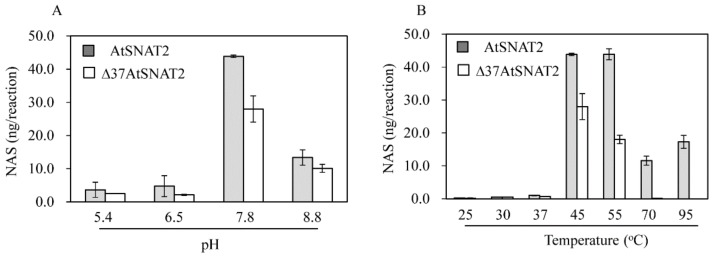
The enzymic activity of recombinant AtSNAT2. (**A**) The activity at various pH values. (**B**) The activity at various temperatures. Enzyme activity was measured by assaying *N*-acetylserotonin (NAS) production by 0.5 μg recombinant protein from 0.5 mM serotonin for 1 h under various conditions.

The pH and temperature optima in terms of enzymic activity were determined. Figure 4 shows that the total SNAT activity of the full-length protein was greater than that of the truncated protein under all conditions tested. The optimal pH values in terms of activity were pH 7.8 for both proteins, followed by pH 8.8 (Figure 4A). SNAT enzyme activity was 10-fold lower in the pH range pH 5.4–6.5 than at pH 7.8. Optimal SNAT enzyme activity at relatively high pH values is characteristic of other plant SNAT2 enzymes [30,31]. The optimal temperatures for activity were 45 ℃ and 55 ℃ for AtSNAT2, but only 45 ℃ for Δ37AtSNAT2. Surprisingly, AtSNAT2 retained 30% of its peak activity even at 95 ℃; Δ37AtSNAT2 exhibited no activity at that temperature (Figure 4B). Neither the truncated rice nor grape enzyme exhibited any activity at 95 ℃ [30,31]. In contrast, rice Δ83SNAT1 exhibited enzyme activity comparable to that of AtSNAT2 at 95 ℃ [35], implying that plant SNAT enzymes are more thermostable than other melatonin biosynthetic enzymes such as ASMT [35].

**Figure 5:**
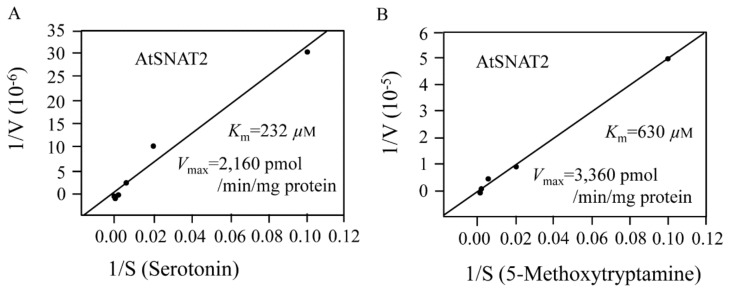
The Michaelis–Menten kinetics of recombinant AtSNAT2. (**A**) The *K*_m_ and *V*_max_ values with serotonin as substrate. (**B**) The *K*_m_ and *V*_max_ values with 5-methoxytryptamine (5-MT) as substrate. *K*_m_ and *V*_max_ values were determined using Lineweaver–Burk plots. The enzymatic products (*N*-acetylserotonin and melatonin) were assayed using high-performance liquid chromatography (HPLC).

**Figure 6:**
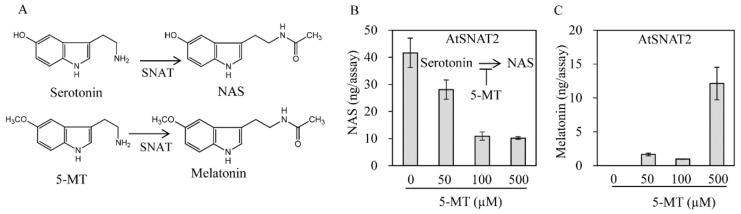
The effect of 5-MT on SNAT enzyme activity. (**A**) A schematic of the SNAT enzymic reaction. (**B**) Inhibition of NAS synthesis by 5-MT. (**C**) Melatonin production in the presence of serotonin (0.5 mM) and varying levels of 5-MT. All assays were performed in the presence of 0.5 μg AtSNAT2, 0.5 mM serotonin, and various levels of 5-MT. The data are the means of three replicates.

In assays performed at the optimal pH and temperature, we next determined *K*_m_ and *V*_max_ values (Figure 5). The *K*_m_ values for NAS and 5-MT were 232 and 630 μM, respectively. The *V*_max_ values for NAS and 5-MT were 2160 and 3360 pmol/min/mg protein, respectively. The catalytic efficiency (*V*_max_/*K*_m_) for NAS was 9.3 and that for 5-MT was 5.3, indicating that AtSNAT favors NAS synthesis from serotonin rather than melatonin synthesis from 5-MT. The effects of 5-MT on enzyme activity were assessed by measuring the conversion of serotonin to NAS in the presence of 5-MT. Figure 6 shows that NAS production (from 500 μM serotonin) was inhibited by the addition of 50 μM 5-MT, and further fell fourfold when 100 μM of 5-MT was added. The decrease in NAS production was not proportional to the 5-MT concentration. Notably, the NAS and melatonin production levels were similar when the substrate levels were 500 μM, implying that the affinity of SNAT for serotonin and 5-MT is similar when substrate levels are high.

**Figure 7:**
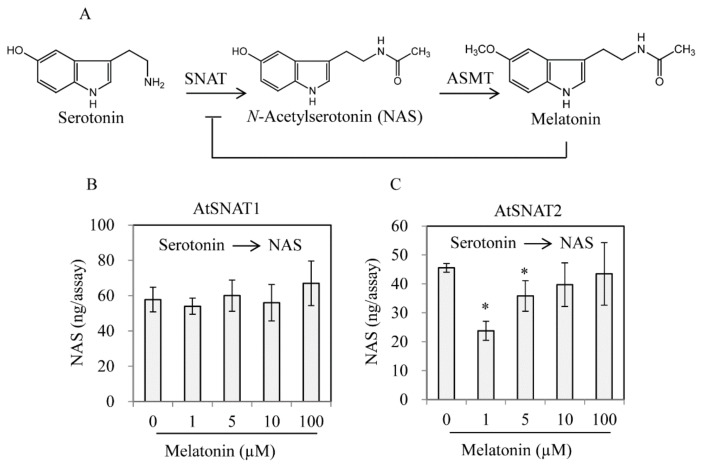
Effect of melatonin on SNAT enzyme activity. (**A**) A schematic of the enzymic reaction. (**B**) Melatonin-mediated inhibition of NAS synthesis by recombinant AtSNAT1. (**C**) Melatonin-mediated inhibition of NAS synthesis by recombinant AtSNAT2. AtSNAT1 was prepared as described previously [28]. The SNAT enzyme assay was performed in the presence of 0.5 μg AtSNAT2, 0.5 mM serotonin, and various levels of melatonin. The data are the means of three replicates. Asterisks (*) denote significant differences compared to the Col-0 controls (Student’s *t*-test; *p* < 0.05).

We explored whether AtSNAT1 [28] and AtSNAT2 activity was feedback-regulated by melatonin. Recombinant AtSNAT2 activity was inhibited by addition of 1 μM melatonin to an extent twofold that of the control (Figure 7), but AtSNAT1 activity was not affected by any tested concentration of melatonin, implying that the modes of action of the SNAT isoenzymes differ.

### 3.4. AtSNAT2 Expression and the Phenotype of the snat2 Knockout Arabidopsis Mutant

**Figure 8:**
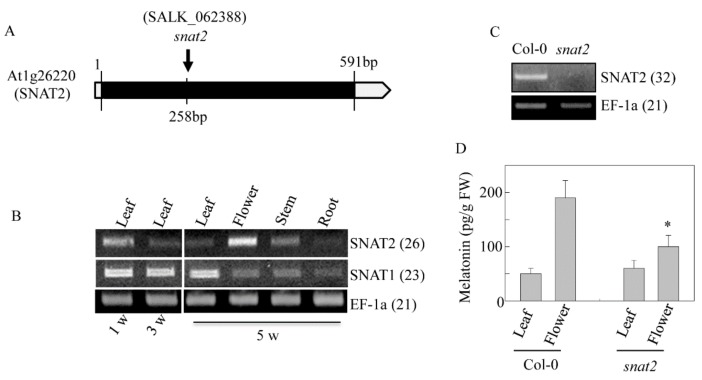
A schematic of *Arabidopsis snat2* knockout and *SNAT2* mRNA expression. (**A**) Genomic structure of the *Arabidopsis SNAT2* gene (At1g26220). Exons and introns are denoted by solid and open boxes, respectively. The location of the T-DNA insertion is arrowed (not to scale). (**B**) Tissue-specific expression levels of *AtSNAT1* and *AtSNAT2* as revealed by RT-PCR. (**C**) RT-PCR analysis of *SNAT2* in wild-type Col-0 and *snat2* mutant. (**D**) Melatonin levels in leaves and flowers. The numbers in parentheses are the PCR cycle numbers. *EF-1a* served as a loading control, as described previously [4]. An asterisk (*) indicates a significant difference compared to the wild-type Col-0 (Student’s *t*-test; *p* < 0.05).

**Figure 9:**
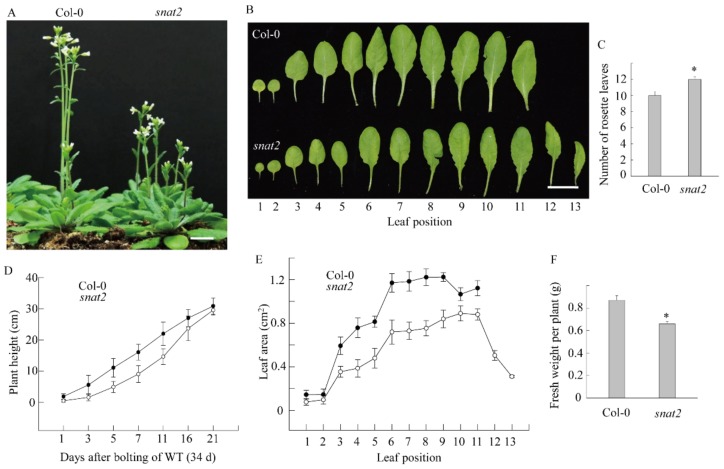
Delayed flowering of the *Arabidopsis snat2* knockout mutant. (**A**) Phenotypes at flowering. (**B**) The rosette leaf phenotype. (**C**) The rosette leaf numbers of the wild-type Col-0 and the *snat2* mutant. (**D**) Stem lengths over time and a comparison of rosette leaf areas. (**E**) Leaf lengths over time. (**F**) Biomass values (n = 10). Stem lengths were measured after the wild type commenced bolting (about 34 d after germination). Bars: 1 cm. Asterisks (*) denote significant differences compared to the wild-type Col-0 (Student’s *t*-test; *p* < 0.05).

To explore directly whether AtSNAT2 engages in melatonin synthesis and the physiological role played by melatonin, we investigated a T–DNA insertional *snat2* knockout mutant (SALK_062388) (Figure 8A). We first measured *SNAT1* and *SNAT2* transcript levels in various tissues via RT-PCR analysis. *SNAT1* mRNA levels were highest in mature leaves; *SNAT2* mRNA was abundant in flowers and young leaves; the two isogenes thus exhibited different expression patterns (Figure 8B). RT-PCR of material from one-week-old leaves showed that *SNAT2* mRNA was absent in the mutant (Figure 8C). Melatonin levels were 50% lower in flowers of the *snat2* mutant than in those of the wild-type Col-0, but the melatonin levels of leaves did not differ, implying that *SNAT2* expression is tissue-specific in terms of both mRNA and melatonin synthesis (Figure 8D). In line with this implication, the *snat2* mutant exhibited delayed flowering, as evidenced by increased leaf numbers (Figure 9A–C). During bolting, the height of the snat2 mutant was less than that of the wild type, but the mutant attained the wild-type height eventually (Figure 9D). The leaf area of the snat2 mutant was less than that of the wild type, reducing the biomass of the mutant (Figure 9E,F).

**Figure 10:**
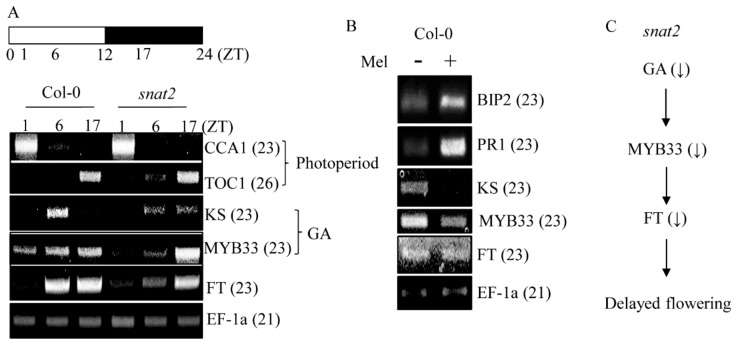
The daily rhythms of flowering-related gene expression. (**A**) Daily rhythms of 4-week-old leaves as revealed by RT-PCR. (**B**) Gene expression levels after addition of exogenous melatonin. (**C**) A model of delayed flowering in the *snat2* mutant. *EF-1a* served as the loading control. Fully mature leaves of 4-week-old wild-type Col-0 *Arabidopsis* were infiltrated with 1 μM melatonin and harvested 3 h later. Water served as the control.

To define the flowering pathway affected by the *snat2* mutation, we used RT-PCR to measure gene expression levels. As shown in Figure 10, the expression levels of photoperiod pathway-related flowering genes, including *Circadian Clock Associated 1* (*CCA1*) and *Timing of CAB expression 1* (*TOC1*), were similar in the mutant and wild type over the 24 h day/night cycle (Figure 10A). However, a key gene involved in GA biosynthesis, *ent-kaurene synthase* (*K*S), was significantly suppressed, whereas GA-responsive negative transcription factor *MYB33* was enhanced in the mutant compared to the wild type, implying that changes in the GA-related pathway (and not the photoperiod pathway) explained the delayed flowering of the *snat2* mutant. Reductions in GA synthesis and enhanced *MYB33* expression decreased levels of *FT* florigen, delaying flowering (Figure 10C). We explored whether flowering-related gene expression was affected by exogenous melatonin (1 μM) that infiltrated leaves over 2 h. As shown in Figure 10B, the expression levels of melatonin-induced representative marker genes such as *BIP2* and *PR1* increased markedly [4], but melatonin suppressed expression of the *KS*, *MYB33*, and *FT* genes, consistent with a previous finding that exogenous melatonin delays flowering [39]. *KS*, *MYB33*, and *FT* suppression by exogenous melatonin was in sharp contrast with such suppression in the *snat2* mutant, indicating that exogenous melatonin did not mimic the effects of endogenous melatonin on flowering.

## 4. Discussion

Given the many available *Arabidopsis* T-DNA knockout mutants, it is simple to explore the roles played by genes of interest [40], including those involved in melatonin metabolism. Melatonin is biosynthesized from tryptophan in four steps catalyzed by tryptophan decarboxylase (TDC), tryptamine 5-hydroxylase (T5H), SNAT, and ASMT [25]. The SNAT-catalyzed reaction is the penultimate step; the *SNAT* gene family plays pivotal roles in terms of melatonin synthesis regulation in both plants and animals [41]. Rice *SNAT1* was first cloned in 2013 [37], followed by *SNAT2* [30]; orthologs exist in all plant taxa explored [27,30]. Both SNAT1 and SNAT2 are nucleus-encoded chloroplast proteins involved in melatonin synthesis. It is known that all plant SNAT proteins explored to date were located in chloroplasts [27,30,31,35]. However, single transgenic rice plants differ in terms of phenotype and stress response. Rice plants overexpressing *SNAT1* are resistant to abiotic stressors such as senescence [21], while *SNAT1* suppression increased the susceptibility of rice plants to cold [42]. However, *SNAT2* suppression conferred tolerance to several abiotic stressors, including both cold and heat [43], and was associated with a novel dwarf-like phenotype combined with erect leaves [10]. Thus, the two *SNAT* isogenes mediate different physiological responses in terms of melatonin synthesis in plants.

Recently, the grape *SNAT2* gene was cloned and characterized [31]. The grapevine SNAT2 is 55% identical (in terms of their amino acid sequences) to rice SNAT2. The optimal pH and temperature for grape and rice SNAT2 activity are similar, but the catalytic efficiency (*V*_max_/*K*_m_) of the grape enzyme is less than that of the rice enzyme [30]. Transgenic *Arabidopsis* overexpressing grape *SNAT2* exhibit enhanced melatonin synthesis and increased resistance to powdery mildew (compared to the wild type) [31]. However, no knockout mutant of grape *SNAT2* has yet been reported. Our *Arabidopsis SNAT2* knockout mutant (*snat2*) clearly exhibited delayed flowering (Figure 9), as confirmed via molecular genetic analysis. Thus, melatonin affects plant flowering to some extent, but does not delay flowering markedly.

In contrast to the effects of the *snat2* mutation, exogenous melatonin retarded *Arabidopsis* flowering by elevating the level of DELLA protein, the master regulator of GA production, in turn suppressing the expression of flowering genes, including *SUPPRESSOR OF OVEREXPRESSION OF CONSTANT 1* (*SOC1*) and *FLOWERING LOCUS T* (*FT*) [39]. These contradictory results are explained by the fact that high-dose melatonin (0.5 mM) retards the growth of *Arabidopsis* seedlings. The melatonin biosynthetic mutant *snat1* also exhibited delayed flowering (data not shown). We are currently studying the *snat1* mutant in detail.

We found, for the first time, that *SNAT2* was involved in melatonin synthesis in *Arabidopsis* and was abundantly expressed in flowers. The *snat2* mutant produced less melatonin than did the wild type, in turn affecting GA biosynthesis and *FT* expression, both of which regulate flowering. We suggest that the reduced melatonin levels are associated with less GA synthesis, and compromise *FT* expression, in turn inhibiting flowering. The mechanism by which melatonin regulates GA synthesis remains to be investigated.

## 5. Conclusions

Melatonin plays roles in both plant growth and defense. Serotonin *N*-acetyltransferase (SNAT) catalyzes the formation of *N*-acetylserotonin (NAS) from serotonin; NAS is then converted to melatonin. Plants contain two *SNAT* isogenes, which exhibit low-level amino acid homology. SNAT is the penultimate enzyme of melatonin biosynthesis. This is the first study to characterize a *snat2* mutant genetically. The mutant exhibited delayed flowering; this is the first genetic indication that melatonin is involved in flowering.
